# Coexisting Primary Biliary Cholangitis and Diffuse Large B-Cell Lymphoma: A Case Report and Literature Review

**DOI:** 10.7759/cureus.79508

**Published:** 2025-02-23

**Authors:** Cheng Ma, Xiaoqian Zhang, Hui Yang

**Affiliations:** 1 Department of Infectious Diseases, Heping Hospital Affiliated to Changzhi Medical College, Changzhi, CHN; 2 Department of Infectious Diseases, First Hospital of Shanxi Medical University, Taiyuan, CHN

**Keywords:** autoimmune diseases, diffuse large b-cell lymphoma, liver damage, pancytopenia, primary biliary cholangitis

## Abstract

It is recognized that autoimmune diseases such as primary Sjogren's syndrome and rheumatoid arthritis have an increased risk of developing lymphoma, particularly non-Hodgkin's lymphoma (NHL). The progression of primary biliary cholangitis (PBC), a chronic autoimmune liver disease, to lymphoma on this basis has rarely been reported. This article describes a 67-year-old female patient who developed diffuse large B-cell lymphoma (DLBCL) one year after the diagnosis of PBC. She had experienced a transient improvement after receiving treatment but died as a result of infections and other related problems triggered by further immune suppression after chemotherapy. We also reviewed the relevant literature to assess the correlation between PBC and lymphoma risk.

## Introduction

Primary biliary cholangitis (PBC) is a chronic, autoimmune, cholestatic disease with clinical manifestations of fatigue, skin pruritus, jaundice, and other symptoms. Its etiology and pathogenesis may be related to immune disorders caused by genetic and environmental factors [1]. Lymphoma is one of the common malignant tumors of the hematological system, and direct involvement of the liver by tumor cells, extrahepatic bile duct obstruction due to enlarged lymph nodes, and liver injury caused by drug therapy are more common, while progression of chronic liver disease to lymphoma is relatively rare [2]. The association between PBC and lymphoma is not coincidental, and it may increase the risk of developing lymphoma [3]. Here, we report a case of PBC combined with diffuse large B-cell lymphoma (DLBCL), including a review of the relevant literature.

## Case presentation

History and physical examination

A 67-year-old woman had intermittent fatigue and abdominal bloating without obvious cause one year previously; she was diagnosed with PBC in several hospitals and began treatment with ursodeoxycholic acid. Two weeks previously, she noticed worsening fatigue and abdominal bloating, accompanied by drowsiness, irritability, nausea, dyspnea, and other discomforting symptoms. Relevant examinations performed at the local hospital revealed ascites, abdominal infection, esophageal varices, hepatic encephalopathy, and various complications. After receiving symptomatic treatment such as hepatoprotection, anti-infection, deamination, and nutritional support, the patient's consciousness disorder and fatigue improved. However, she continued to experience abdominal distension and dyspnea and was admitted to our hospital for further diagnosis and treatment.

On admission, the patient's physical examination revealed yellow staining of the skin, mucous membranes, and sclera. A swollen lymph node, about 3 cm in diameter, rigid, and with poor mobility, was palpable on the right side of the neck. The spleen was palpable 1 cm below the left subcostal margin with a medium texture; the liver was not enlarged. Shifting dullness was positive, and concave edema was present in the lower limbs.

Hospital course

Laboratory tests revealed the following results: white blood cells, 1.7×10^9^/L (neutrophils 45.0%, lymphocytes 13.6%, monocytes 38.3%); hemoglobin, 94g/L; platelets count of 27×10^9^/L. Additionally, liver function tests showed: alanine aminotransferase, 20U/L (normal, 7-40U/L); aspartate aminotransferase, 56U/L (normal, 13-35U/L); alkaline phosphatase 212U/L (normal, 50-135U/L); gamma-glutamyl transferase 139U/L (normal, 7-45U/L); a total bilirubin level of 74.4μmol/L (normal, 0-23μmol/L); bile acids, 354.3μmol/L (normal, 0-10μmol/L); and an albumin level of 34.1g/L (normal, 40-55g/L). Coagulation tests showed a prothrombin time of 18 seconds (control, 10-14 seconds) and prothrombin activity of 46.1% (normal, 70-130%). Inflammatory markers showed procalcitonin, 0.768ng/ml (normal, 0-0.05ng/ml); and C-reactive protein, 53.71mg/L (normal, 0-8.0mg/L). Antinuclear antibody and antimitochondrial antibody M2 subtype were positive, while antibodies for hepatitis B and C viruses were negative. Important laboratory indicators during hospitalization are detailed in Table 1. Ascite pathology showed inflammatory cells, mesothelial cells, and histiocytes. 

**Table 1:** Laboratory investigations

Parameters	Patient values					Reference range
	Admission	1 week	3 week	1 month	Pre-death	
Hemoglobin, g/L	94.0	75.2	90.0	92.0	97.0	115-150
White cell count, ×10^9^/L	1.7	1.2	19.1	2.4	11.3	3.5-9.5
Platelets, ×10^9^/L	27	22	40	23	64	125-350
Alanine aminotransferase, U/L	20	17	51	59	58	7-40
Aspartate aminotransferase, U/L	56	65	71	59	76	13-35
Alkaline phosphatase, U/L	212	202	239	254	202	50-135
Gamma-glutamyl transferase, U/L	139	132	103	165	132	7-45
Total bilirubin, umol/L	74.4	65.5	114.1	194.4	132.6	0-23
Total bile acid, umol/L	354.3	560.0	239.5	411.0	288.9	0-10
Albumin, g/L	34.1	30.3	32.7	30.9	24.3	40-55
Blood urea nitrogen, mmol/L	6.6	9.8	17.5	42.2	51.8	2.8-7.6
Creatinine, umol/L	54.0	75.0	71.2	79.8	238.4	41-81
Prothrombin time, s	18.0	18.0	17.1	19.5	19.9	10-14
prothrombin activity, %	46.1	47.1	49.9	40.7	38.2	70-130
International normalized ratio	1.56	1.66	1.57	1.69	1.71	0.8-1.2
Procalcitonin, ng/ml	0.77	1.06	0.49	2.82	6.14	0-0.05
C-reactive protein, mg/L	53.71	69.55	37.72	89.17	190.08	0-8.0
(1,3)-beta-D-glucan test, pg/mL	-	58.17	44.80	235.94	253.71	＜70
Galactomannan test, s/co	-	0.07	0.39	5.07	4.50	＜0.5
Anti-nuclear antibodies	Positive	-	-	-	-	-
Anti-mitochondrial antibodies	Positive	-	-	-	-	-
Hepatitis B	Negative	-	-	-	-	-
Hepatitis C	Negative	-	-	-	-	-

**Figure 1:**
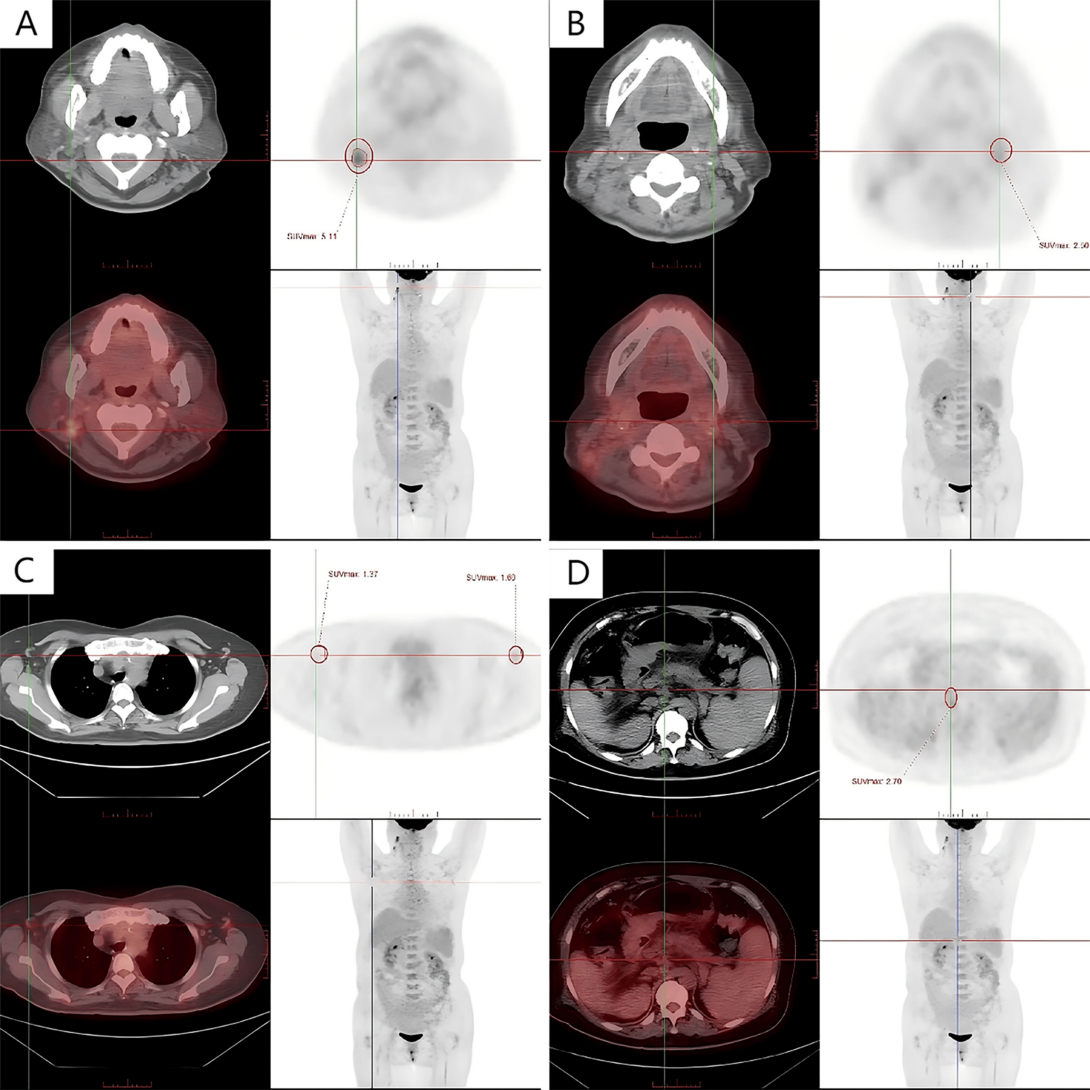
PET/CT images. (A) Multiple enlarged lymph nodes in region II of the right neck, larger about 1.5cm x 1.4cm, SUVmax=5.11 (red circle). (B) Multiple lymph node formations were seen in the left cervical region I-V, some with increased metabolism, SUVmax=5.11 (red circle). (C) Lymph nodes with slightly higher metabolism were seen in both axillae, the sizes were about 1.4cm x 0.8cm, SUVmax=1.37-1.69 (red circles). (D) Multiple lymph nodes were seen slightly enlarged in the hepatoportal region and hepatogastric ligament, some of them mildly metabolized, maximum about 1.2 cm x 0.9 cm, SUVmax=2.70 (red circle).

**Figure 2:**
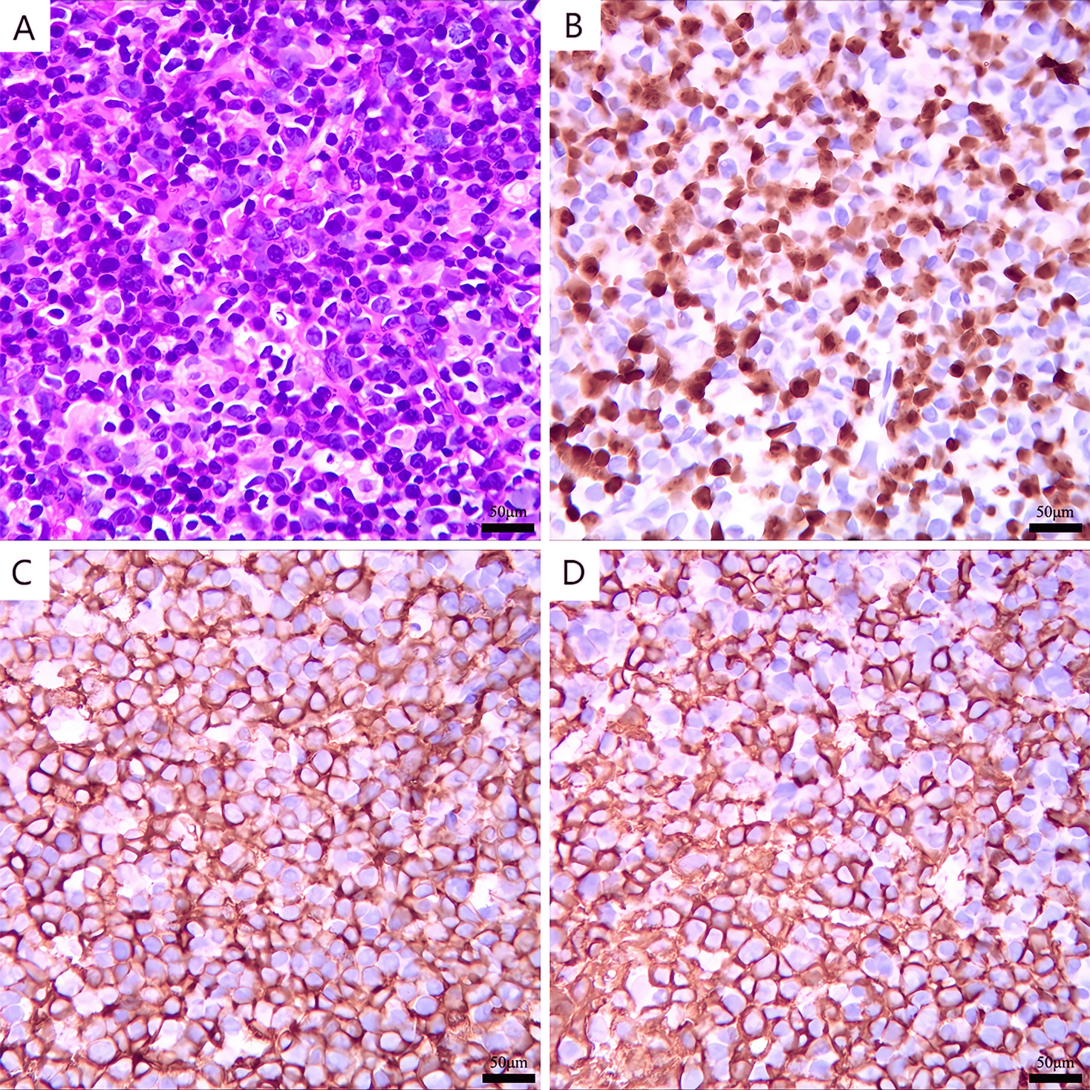
Puncture specimen of a right cervical lymph node. (A)  Diffuse proliferation of tumor cells (HE staining, ×400). (B) Ki-67 (70%+) (immunohistochemistry, ×400). (C) CD19 (+) (immunohistochemistry, ×400). (D) CD20 (+) (immunohistochemistry, ×400).

Systemic imaging showed enlarged lymph nodes in the bilateral neck I-V region, bilateral armpit, hepatic portal, and hepatogastric ligament, with partial metabolic elevation (Figure 1). Lymph node puncture pathology: (right neck) lymph node diffuse large B-cell lymphoma, non-specific type, non-germinal center B-cell origin (Hans model) (Figure 2); immunohistochemistry showed: CD19 (+), CD20 (+), CD3 (-), CD5 (-), CD10 (-), CD21 (-), CD30 (5%+), KI67 (70% +), MYC (10%+), BCL2 (20%+), BCL6 (-), MUM1 (+), P53 (30%+), CD79b (-), in situ hybridization result: EBER (-). 

The hematologist diagnosed the patient with non-GCB DLBCL, clinical stage IV B (involving the bilateral neck, bilateral armpit, and hepatic portal lymph node with suspicious bone marrow and peritoneal involvement). Low-dose dexamethasone was prescribed to reduce tumor burden and the patient was treated with rituximab, cyclophosphamide, doxorubicin, vincristine, and prednisone (R-CHOP). During chemotherapy, her mental health and appetite improved significantly, the proportion of peripheral blood monocytes decreased to normal, and the pancytopenia and coagulation abnormalities improved considerably. However, five days after the end of chemotherapy, the patient developed chemotherapy-induced bone marrow suppression, which showed a new decrease in blood cells and an abnormally prolonged clotting time. Stool culture showed *Candida *80% and the serum (1,3)-β-D-glucan (G test) level was elevated to 235.94 pg/ml, suggesting aggravation of abdominal and pulmonary fungal infection. Her condition remained critical despite anti-infective treatment with linezolid and meropenem combined with caspofungin, and she eventually died of septic shock.

## Discussion

For our literature review, we selected 11 case reports (Table 2); all of these cases developed malignant lymphoma based on PBC and described the treatment process in detail. The vast majority of patients were reported to have a stable course of disease after being dosed with ursodeoxycholic acid. Some patients had a history of autoimmune diseases other than PBC or other conditions, such as Sjogren's syndrome, pulmonary fibrosis, transverse myelitis, and bronchial asthma, and were given different doses of steroid hormones to control the condition and relieve symptoms. Each of these cases carried out a physical examination of these patients and described clinical symptoms such as varying degrees of enlarged lymph nodes, enlarged liver and spleen, and cutaneous lesions. All patients completed biopsy and histopathology of different sites, and 10 cases were diagnosed with NHL, including DLBCL in five [4-8], mucosa-associated lymphoid tissue (MALT) lymphoma in four [9-12], and cutaneous T cell lymphoma in one [13]. In eight cases of NHL in extra-nodal sites, the most common site was the liver [6,7,9,11], followed by the stomach [8], rectum [10], eyelids [12], and skin [13]. One case was diagnosed as Hodgkin's lymphoma (HL) [14], and its histological subtype was mixed cellularity Hodgkin's disease. All patients received appropriate therapy after the diagnosis of lymphoma, except two patients who gave up therapy [7,8]. Seven patients were followed up in a short period, three were stable [7,11,13], two were in complete remission [10,12], and two died due to pulmonary infection and deterioration [4,6].

**Table 2:** Reported cases of primary biliary cholangitis that later developed or simultaneously presented with lymphoma, categorized by the first author Interval refers to the years between diagnosis of primary biliary cholangitis and diagnosis of lymphoma. ^a^ alive at the end of the follow-up period. Abbreviations: NHL, non-Hodgkin lymphoma; HL, Hodgkin lymphoma; DLBCL, diffuse large B-cell lymphoma; MALT, mucosa-associated lymphatic tissue lymphoma; CTCL, cutaneous T cell lymphoma; MCCHL, mixed cellularity classical Hodgkin lymphoma; CT, chemotherapy; RT, radiation therapy; PUVA, psoralen plus ultraviolet A photochemotherapy

Authors	Gender	Age	Medical history	Interval (in years)	Presentation of lymphoma	Type of lymphoma	Histology subtype	Treatment	Follow-up period (in years）
Kanellopoulou et al. [4]	Female	75	Pulmonary fibrosis	10	Lymphadenopathy, hepatomegaly, splenomegaly	NHL	DLBCL	CT	0.4
Wakatsuki et al. [5]	Female	53	Sjögren's syndrome, transverse myelitis	3	Lymphadenopathy	NHL	DLBCL	No treatment	Not reported
Goldin et al. [6]	Female	52	Not significant	4	No symptoms	NHL	DLBCL	CT	0.2
Sato et al. [7]	Female	55	Sjögren's syndrome	14	Hepatomegaly, splenomegaly	NHL	DLBCL	No treatment	1^a^
Feng et al. [8]	Female	40	Not significant	5	Splenomegaly	NHL	DLBCL	No treatment	Not reported
Nakayama et al. [9]	Female	80	Not significant	Simultaneous	No symptoms	NHL	MALT	CT	Not reported
Kawashima et al. [10]	Female	83	Sjögren's syndrome	21	Splenomegaly	NHL	MALT	Rituximab	0.3^a^
Ye et al. [11]	Female	57	Not significant	—	No symptoms	NHL	MALT	Surgery	0.8^a^
Hahn et al. [12]	Female	61	Sjögren's syndrome	Simultaneous	Lymphadenopathy	NHL	MALT	RT	0.7^a^
Stroehmann et al. [13]	Female	51	Sjögren's syndrome, pulmonary fibrosis	5	Cutaneous lesion, hepatomegaly	NHL	CTCL	PUVA	2^a^
Viteri et al. [14]	Female	59	Bronchial asthma	2	Lymphadenopathy, splenomegaly	HL	MCCHL	CT	Not reported
Present study	Female	67	Not significant	1	Lymphadenopathy, splenomegaly	NHL	DLBCL	CT	Not reported

PBC is the apoptotic necrosis of intrahepatic biliary epithelial cells caused by the breakdown of hepatic immune tolerance and aberrant activation of the immune system in individuals with genetic susceptibility under environmental triggers. Studies have shown that a variety of autoimmune diseases such as primary Sjogren's syndrome, rheumatoid arthritis, systemic lupus erythematosus, autoimmune hemolytic anemia, and Hashimoto's thyroiditis, etc., are prone to progress to lymphoma, especially DLBCL and marginal zone lymphoma [15], and the pathogenesis of these may be related to the body's persistent chronic inflammation, antigenic drive, lymphocyte activation, immune imbalance, and immune-suppressive treatment [16]. Floreani et al. identified advanced histological staging and extrahepatic autoimmune disease as risk factors for the development of extrahepatic malignancy in PBC [17], but the mechanism of the development of independent progression of PBC to lymphoma is currently inconclusive and needs to be clarified by meticulous immunological and pathological studies. The discovery of lymphoma in our patient one year after the diagnosis of PBC suggests that PBC patients may be at increased risk of developing lymphoma. Panjala et al. followed up on 2,192 patients with PBC and observed that the incidence of lymphoma was 0.6% (13 cases) and that the majority of patients (54%) had PBC before their lymphoma was diagnosed [3]. In a cohort study involving two European centers, the overall cancer incidence in PBC patients was found to be similar to that of the general population, but lymphoma was significantly more prevalent in PBC patients than in the normal population [17].

Lymphoma is a group of malignant tumors originating from the lymphohematopoietic system, most commonly found in middle-aged and elderly people, and can be divided into HL and NHL according to histopathology, with the latter accounting for approximately 90% of all lymphomas. DLBCL is the most common aggressive lymphoma and its predisposing factors include the use of drugs that produce molecular aberrations, and congenital or acquired immunodeficiency states. Research has shown that about one-third of DLBCL patients have extranodal involvement of lymph nodes and that common sites include the bone marrow, gastrointestinal tract, soft tissues of the skin, central nervous system, liver, lungs, etc. Specific extranodal sites involved in DLBCL are associated with unique clinical features, and multiple extranodal lesion involvement may lead to worse survival outcomes and prognosis [18]. In our case, the primary disease of the patient, PBC, is a slowly progressive disease with a natural course of about 10-20 years from onset to progression to cirrhosis. Within one year of onset, this patient developed serious complications of cirrhotic decompensation such as hepatic encephalopathy and abdominal infection. The disease progressed rapidly, in addition to the individual differences in PBC disease itself, the direct infiltration of abnormal lymphoma cells of the DLBCL, bile duct obstruction due to lymph node proliferation in the hepatic portal region, paraneoplastic syndrome, and the occurrence of opportunistic infections led to the exacerbation of liver damage, as the patient developed complex manifestations of more advanced liver disease. During the treatment, the patient's whole blood cell progressive hypocellularity was particularly prominent. Combined with the patient's history of liver disease, hypersplenism is not sufficient to cause severe hypocellularity of the whole blood cell, considering the degree of liver disease progression. We consider that lymphoma invasion of the bone marrow, hemophagocytic syndrome caused by lymphoma or severe infection, and various overlapping effects make pancytopenia difficult to correct; the risk of infection and hemorrhage is also increased.

The treatment of lymphoma is a comprehensive model based on medical treatments such as chemotherapy, targeted therapy, immunotherapy, and the combination of the anti-CD20 monoclonal antibody rituximab and CHOP chemotherapy has become the standard therapy for patients with DLBCL [19]. In the case of this patient with poor treatment of decompensated cirrhosis with ascites, combined with the accelerated progression of liver disease, severe hypocellularity of complete blood cells, and the confirmatory basis of lymph node biopsy, the possibility of lymphoma invasion to the liver could not be excluded. However, the inability to conduct a liver biopsy due to the patient's poor coagulation function and severely low platelet levels is a limitation of this case.

After the first cycle of chemotherapy, the patient's general conditions such as spirit and appetite improved significantly, as well as the ascites, pancytopenia, and coagulation abnormality improved, suggesting that the treatment was effective. However, for patients with underlying PBC, poor liver function, and extremely low immunity, drug-related liver injury caused by chemotherapy may lead to further liver function deterioration, while infection-related problems brought about by further immune suppression such as pulmonary infection, abdominal infections, etc., and secondary infections from fungi and other pathogens are an important factor ultimately leading to the death of the patient. Patients with poor prognoses, as suggested by the literature, also mostly died due to worsening conditions caused by pulmonary infections [4,6].

## Conclusions

As an autoimmune disease, PBC is strongly associated with malignant lymphoma. The occurrence of lymphoma can exacerbate liver damage and significantly accelerate the disease process in pre-existing liver disease. The cases presented here emphasize that when PBC patients with progressive disease deterioration, unexplained lymph node enlargement, difficult-to-correct severe pancytopenia, or are accompanied by extra-hepatic manifestations, clinicians should be vigilant for early recognition and timely diagnosis of lymphoma, and invite hematologists to be involved in the patient's management and treatment. To maximize the benefit to patients, we believe that routine screening, regular follow-up, and early intervention if necessary are very important for PBC patients at risk of lymphoma.
